# Trends in all causes and cause specific mortality attributable to ambient particulate matter pollution in China from 1990 to 2019: A secondary data analysis study

**DOI:** 10.1371/journal.pone.0291262

**Published:** 2023-09-08

**Authors:** Yingying Liu, Mengmeng Yan

**Affiliations:** 1 Department of Health Management & Institute of Health Management, Sichuan Provincial People’s Hospital, University of Electronic Science and Technology of China, Chengdu, China; 2 Chinese Academy of Sciences Sichuan Translational Medicine Research Hospital, Chengdu, China; 3 School of Healthcare and Technology, Chengdu Neusoft University, Chengdu, China; Atal Bihari Vajpayee Institute of Medical Sciences & Dr Ram Manohar Lohia Hospital, INDIA

## Abstract

**Background:**

Particularly fine particulate matter (PM2.5) has become a significant public health concern in China due to its harmful effects on human health. This study aimed to examine the trends in all causes and cause specific morality burden attributable to PM2.5 pollution in China.

**Methods:**

We extracted data on all causes and cause specific mortality data attributable to PM2.5 exposure for the period 1990–2019 in China from the Global Burden of Disease 2019. The average annual percent change (AAPC) in age-standardized mortality rates (ASMR) and years of life lost (YLLs) due to PM2.5 exposure were calculated using the Joinpoint Regression Program. Using Pearson’s correlation, we estimated association between burden trends, urban green space area, and higher education proportions.

**Results:**

During the period 1990–1999, there were increases in mortality rates for All causes (1.6%, 95% CI: 1.5% to 1.8%), Diabetes mellitus (5.2%, 95% CI: 4.9% to 5.5%), Encephalitis (3.1%, 95% CI: 2.6% to 3.5%), Ischemic heart disease (3.3%, 95% CI: 3% to 3.6%), and Tracheal, bronchus and lung cancer (5%, 95% CI: 4.7% to 5.2%). In the period 2010–2019, Diabetes mellitus still showed an increase in mortality rates, but at a lower rate with an AAPC of 1.2% (95% CI: 1% to 1.4%). Tracheal bronchus and lung cancer showed a smaller increase in this period, with an AAPC of 0.5% (95% CI: 0.3% to 0.6%). In terms of YLLs, the trends appear to be similar.

**Conclusion:**

Our findings highlight increasing trends in disease burden attributable to PM2.5 in China, particularly for diabetes mellitus, tracheal, bronchus, and lung cancer.

## Introduction

Air pollution, particularly fine particulate matter (PM2.5), has emerged as a significant public health concern in recent years due to its detrimental impact on human health [1, 2]. PM2.5 comprises particles with a diameter of 2.5 micrometers or less and can deeply penetrate the respiratory system, leading to a range of health issues, including respiratory and cardiovascular diseases, as well as cancer [3–5]. The mechanism through which PM2.5 particulate matter causes damage to the human body involves several pathways [4, 5]. When inhaled, these tiny particles can reach the deepest parts of the lungs, where they can trigger inflammation and oxidative stress. The particles can also carry toxic compounds and heavy metals, which further contribute to cellular damage and the development of diseases. Additionally, PM2.5 can impair lung function, weaken the immune system, and increase the susceptibility to respiratory infections. Additionally, PM2.5 pollution contributes to climate change, affecting agriculture, ecosystems, and global weather patterns [6].

China is currently grappling with severe PM2.5 pollution, with numerous cities experiencing levels that surpass the air quality guidelines set by the World Health Organization (WHO) [7, 8]. Factors such as rapid urbanization, industrialization, and increased energy consumption have contributed to the deterioration of air quality in the country [9]. Major sources of PM2.5 pollution in China include coal combustion, vehicle emissions, industrial processes, and biomass burning [10].

Previous research has explored the relationship between PM2.5 pollution and disease burden. For instance, Burnett et al. (2018) discovered that long-term exposure to ambient PM2.5 was associated with increased mortality risks in a large global population [11]. Chen et al. (2017) reported that PM2.5 pollution was responsible for 1.37 million deaths in China in 2015, accounting for 15.5% of total deaths in the country [12]. Moreover, Wang et al. (2018) demonstrated that PM2.5 pollution contributed to a substantial increase in years of life lost (YLL) in China, particularly due to cardiovascular and respiratory diseases [13].

While these studies have provided valuable insights into the relationship between PM2.5 pollution and disease burden, there is still a need for a comprehensive analysis of the impact of PM2.5 pollution on all-cause and cause-specific disease burden in China using the most recent GBD data.

Assessing the impact of PM2.5 pollution on disease burden in China is of utmost importance in formulating effective policies to combat this pressing public health issue. The objective of this study is to examine trends in all-cause and cause-specific disease burden attributed to PM2.5 pollution in China, utilizing data from the Global Burden of Disease (GBD) project. The findings will provide valuable insights for policymakers and serve as a guide for efforts aimed at enhancing air quality and public health in the country.

## Methods

Data for this study were obtained from two sources. First, the Global Burden of Disease Study 2019 [14] was used to extract data on all-cause and cause-specific mortality rates attributable to PM2.5 exposure for the period 1990–2019 in China. The data download setting included Ambient particulate matter pollution, mortality, Years of life lost, all causes and all level three causes, sex, age-standardized, China, 1990–2019. The specific diseases are as follows: Chronic obstructive pulmonary disease (J41-J44.9), Diabetes mellitus (E10-E10.1, E10.3-E11.1, E11.3-E11.9, P70.2), Diarrheal diseases (A00-A00.9, A02-A02.0, A02.8-A07, A07.2-A07.4, A08-A09.9, K52.1, R19.7), Encephalitis (A83-A86.4, B94.1, F07.1, G04-G05.8, G21.3), Ischemic heart disease (I20-I25.9), Lower respiratory infections (A48.1, A70, B97.4-B97.6, J09-J15.8, J16-J16.9, J20-J21.9, J91.0, P23.0-P23.4, U04-U04.9), Meningitis (A39-A39.9, A87-A87.9, G00.0-G00.8, G03-G03.8), Neonatal disorders (P00-P04.2, P04.5-P05.9, P07-P15.9, P19-P22.9, P24-P29.9, P36-P36.9, P38-P39.9, P50-P61.9, P70-P70.1, P70.3-P72.9, P74-P78.9, P80-P81.9, P83-P84, P90-P94.9, P96, P96.3-P96.4, P96.8), Otitis media (H70-H70.9), Stroke (G45-G46.8, I60-I63.9, I65-I66.9, I67.0-I67.3, I67.5-I67.6, I68.1-I68.2, I69.0-I69.3), Sudden infant death syndrome (R95-R95.9), and Tracheal, bronchus, and lung cancer (C33-C34.9, D02.1-D02.3, D14.2-D14.3, D38.1). The GBD 2019 provides a comprehensive assessment of mortality and health risks associated with air pollution at the global, regional, and national levels, making it a suitable data source for this analysis. Exposure to PM2.5 is defined as the population-weighted annual average mass concentration of particles with an aerodynamic diameter less than 2.5 micrometers in a cubic meter of air.

The second data source is the official data from the National Bureau of Statistics of China [15]. We extracted data on urban green space area for the period 2004–2019 and the number of college graduates or above from 1998 to 2019 from this database. These choices were dictated by the data availability and the specific years for which relevant data were provided by the statistical agency. The National Bureau of Statistics of China is the official statistical agency of the Chinese government, responsible for collecting, processing, and disseminating various types of statistics at the national level. The urban green space area data we selected is based on the annual urban land use statistics provided by the National Bureau of Statistics of China, while the number of college graduates is based on the annual national education development statistics. To estimate the proportion of the higher education population, we divided the number of college graduates or above by the total population at the end of each year.

We employed the Joinpoint Regression Program (National Cancer Institute, version 5.1) to conduct our statistical analysis. This program allowed us to calculate the Average Annual Percent Change (AAPC) in Age-Standardized Mortality Rates (ASMR) and Years of Life Lost (YLLs) attributed to PM2.5 exposure. To assess the temporal patterns of mortality burden trends, we divided the study period from 1990 to 2019 into three distinct time intervals: 1990–1999, 2000–2009, and 2010–2019. Using the Joinpoint Regression Program, we set five joinpoints to identify any significant changes in the trends during these intervals. For each interval, we calculated the AAPC, which provided insight into the average annual percentage change in ASMR and YLLs over the specified time period. To investigate the potential factors influencing the changes in mortality burden trends, we examined the associations between two factors, namely urban green space area and the proportion of the population with higher education, and the mortality rates. To quantify the strength and direction of these relationships, we calculated Pearson correlation coefficients for each pair of variables. We considered correlation coefficients with p-values less than 0.05 as statistically significant, indicating a significant linear relationship between the variables.

Statistical significance was nonoverlapping 95% CIs. Analyses were done using Stata, version 17.0 (USA) and Prism 9 (USA). The source data used in this study can be found in the S1 Table.

## Results

### ASMR in 1990 and 2019

Table 1 presents the ASMR attributable to PM2.5 for various diseases in 1990 and 2019. In 2019, the ASMR for all causes attributed to ambient particulate matter pollution was 81.283 (95% CI: 67.184 to 96.133) deaths per 100,000 population, showing a slight increase compared to 77.150 (95% CI: 38.468 to 126.562) deaths per 100,000 population in 1990. Notably, the ASMR for chronic obstructive pulmonary disease (COPD) significantly decreased from 33.818 (95% CI: 17.029 to 55.691) deaths per 100,000 population in 1990 to 16.593 (95% CI: 12.805 to 21.802) in 2019. Conversely, the ASMR for diabetes mellitus increased from 0.654 (95% CI: 0.291 to 1.156) deaths per 100,000 population in 1990 to 1.736 (95% CI: 1.216 to 2.322) deaths per 100,000 population in 2019. Diarrheal diseases, encephalitis, meningitis, otitis media, sudden infant death syndrome, and upper respiratory infections all had low mortality rates in both 1990 and 2019. The age-standardized YLLs increased slightly between 1990 and 2019. However, there were significant decreases in YLLs rates for COPD and lower respiratory infections, while YLLs rates for diabetes mellitus and ischemic heart disease showed notable increases. In addition, within the gender subgroups, males have higher rates of cardiovascular diseases (68.897, 95% CI: 84.389–53.922) and stroke (40.259, 95% CI: 49.427–31.389) compared to females (37.034, 95% CI: 46.677–28.040 and 21.288, 95% CI: 27.053–15.974, respectively). Additionally, males are more affected by tracheal, bronchus, and lung cancer (13.364, 95% CI: 18.192–9.211) than females (4.991, 95% CI: 6.723–3.500). Conversely, chronic respiratory diseases, including chronic obstructive pulmonary disease, are more prevalent in males (24.168, 95% CI: 30.819–18.410) compared to females (12.083, 95% CI: 17.416–8.661).

**Table 1:** All causes and specific cause age-standardized mortality rates and years of life lost in 1990 and 2019, China.

Causes	ASMR		ASYLLs	
	1990	2019	1990	2019
All causes	77.15(38.468,126.562)	81.283(67.184,96.133)	1703.439(828.372,2824.607)	1512.779(1238.214,1794.255)
Chronic obstructive pulmonary disease	33.818(17.029,55.691)	16.593(12.805,21.802)	505.462(250.295,839.976)	218.33(167.779,286.827)
Diabetes mellitus	0.654(0.291,1.156)	1.736(1.216,2.322)	13.443(5.96,23.923)	31.467(21.905,42.325)
Diarrheal diseases	0.013(0.006,0.023)	0.001(0.001,0.002)	1.183(0.506,2.077)	0.13(0.092,0.179)
Encephalitis	0.001(0,0.002)	0.001(0.001,0.001)	0.079(0.033,0.142)	0.077(0.054,0.115)
Ischemic heart disease	8.631(4.207,14.285)	21.311(17.224,25.67)	181.243(84.866,301.344)	396.626(319.058,482.302)
Lower respiratory infections	7.177(3.376,12.451)	2.731(1.818,3.786)	385.672(175.028,680.845)	66.412(45.093,90.619)
Meningitis	0.005(0.002,0.009)	0.002(0.001,0.003)	0.447(0.199,0.786)	0.175(0.127,0.23)
Neonatal disorders	0.875(0.398,1.551)	0.607(0.458,0.764)	77.79(35.366,137.822)	53.919(40.664,67.891)
Otitis media	0(0,0)	0(0,0)	0.001(0,0.001)	0(0,0)
Stroke	22.595(10.692,38.11)	29.545(23.879,35.224)	457.29(213.832,774.45)	560.947(449.531,671.739)
Sudden infant death syndrome	0.001(0,0.003)	0.002(0.001,0.002)	0.097(0.025,0.27)	0.141(0.066,0.218)
Tracheal, bronchus, and lung cancer	3.377(1.516,5.728)	8.754(6.262,11.573)	80.63(35.634,136.514)	184.541(130.979,246.652)
Upper respiratory infections	0.001(0,0.003)	0(0,0)	0.103(0.009,0.232)	0.015(0.006,0.044)

ASMR = age-standardized mortality rates, ASYLLs = age-standardized years of life lost.

### Trends of ASMR

**Fig 1:**
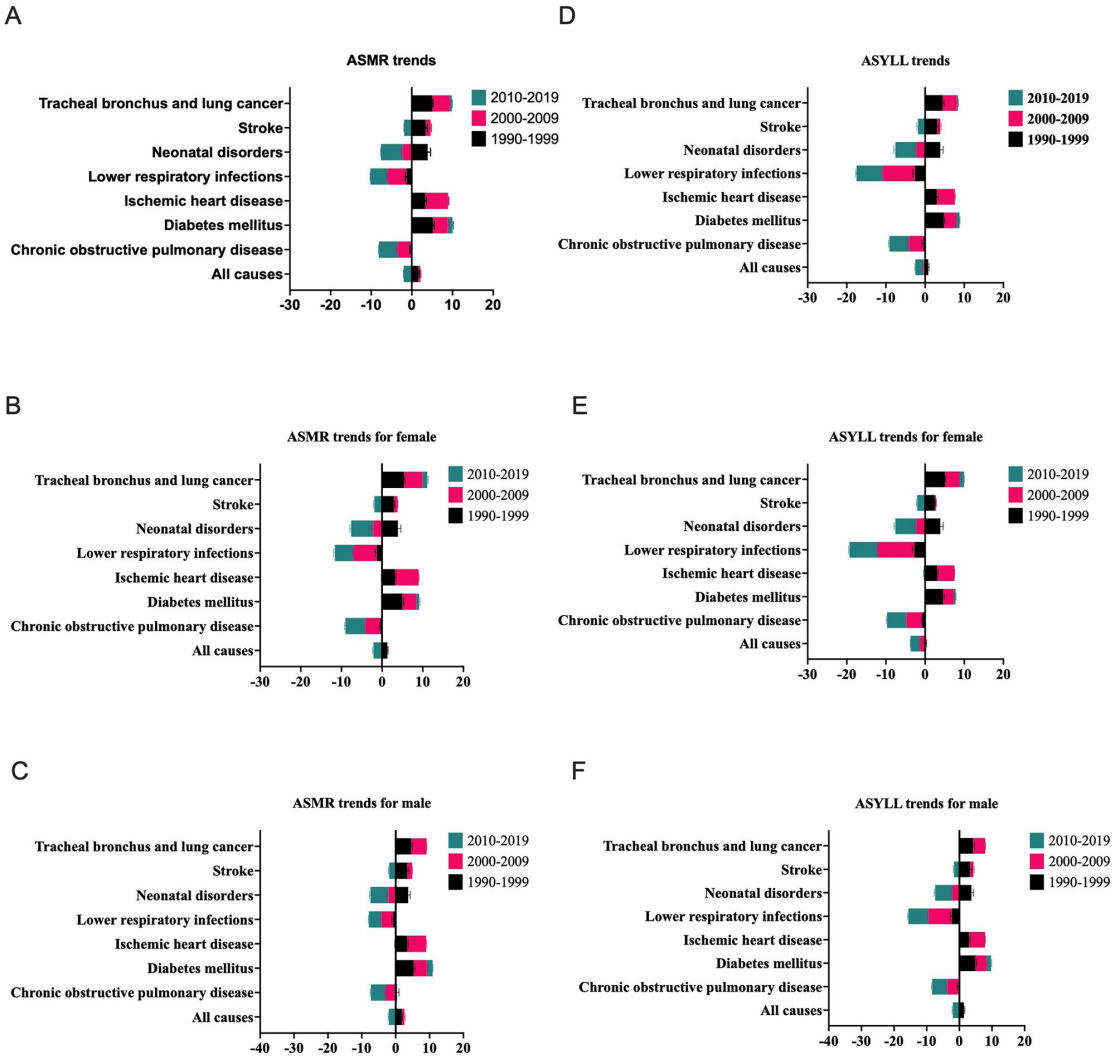
Trends in the burden attributable to PM2.5 for all causes and specific causes in China, by gender and time periods. ASMR: age-standardized mortality rates, YLL: age-standardized years of life lost. Disease 1: All causes, Disease 2: Chronic obstructive pulmonary disease, Disease 3: Diabetes mellitus, Disease 4: Ischemic heart disease, Disease 5: Lower respiratory infections, Disease 6: Neonatal disorders, Disease 7: Stroke, Disease 8: Tracheal bronchus and lung cancer. We utilized joinpoint regression analysis to examine PM2.5 exposure-related disease burden trends from 1990 to 2019. The regression results revealed varying time connection points for different disease types (refer to S2 Table). To facilitate comparative analysis, we calculated the AAPC for three distinct periods: 1990–1999, 2000–2009, and 2010–2019. AAPC serves as a robust indicator of overall trends within each decade, mitigating the impact of short-term fluctuations. A positive AAPC indicates an increasing burden of PM2.5 exposure-related diseases over a decade, while a negative AAPC signifies a decreasing trend.

During the period 1990–1999 (Fig 1 and S2 Table), there were increases in mortality rates for all causes (1.6%, 95% CI: 1.5% to 1.8%), diabetes Mellitus (5.2%, 95% CI: 4.9% to 5.5%), encephalitis (3.1%, 95% CI: 2.6% to 3.5%), ischemic heart disease (3.3%, 95% CI: 3% to 3.6%), and tracheal bronchus and lung cancer (5%, 95% CI: 4.7% to 5.2%). In the 2000–2009 period, the mortality rates continued to increase for diabetes mellitus (3.6%, 95% CI: 3.5% to 3.8%), ischemic heart disease (5.6%, 95% CI: 5.4% to 5.8%), and Tracheal bronchus and lung cancer (4.3%, 95% CI: 4.1% to 4.4%). In the 2010–2019 period, diabetes mellitus still showed an increase in mortality rates, but at a lower rate with an AAPC of 1.2% (95% CI: 1% to 1.4%). Tracheal bronchus and lung cancer showed a smaller increase in this period, with an AAPC of 0.5% (95% CI: 0.3% to 0.6%). In terms of gender subgroups (Fig 1), the trends appear to be similar between males and females.

### Trends of age-standardized YLLs

For YLLs (Fig 1 and S2 Table), the significant increases in AAPC were observed in diabetes mellitus, with an increase of 4.8% (95% CI: 4.6% to 5.0%) during 1990–1999, and 3.1% (95% CI: 3.0% to 3.2%) during 2000–2009, before decreasing to 0.9% (95% CI: 0.7% to 1.0%) during 2010–2019. Similarly, ischemic heart disease experienced an increase in AAPC from 3.0% (95% CI: 2.8% to 3.3%) during 1990–1999 to 4.6% (95% CI: 4.5% to 4.7%) during 2000–2009, followed by a non-significant change in 2010–2019. Additionally, for tracheal, bronchus, and lung cancer under Ambient Particulate Matter Pollution, the AAPC increased from 0.4% (95% CI: 0.2% to 0.6%) during 1990–1999 to 2.5% (95% CI: 2.4% to 2.6%) during 2000–2009, followed by a decrease to 0.8% (95% CI: 0.7% to 0.9%) during 2010–2019. In terms of all causes, the AAPC increased by 0.8% (95% CI: 0.7% to 1.0%) during 1990–1999, followed by a decrease of 0.4% (95% CI: -0.5% to -0.3%) during 2000–2009, and a further decrease of 2.1% (95% CI: -2.2% to -1.9%) during 2010–2019. In terms of gender subgroups (Fig 1), the trends also appear to be similar between males and females. For example, in diabetes mellitus, the AAPC for males increased by 4.9% (95% CI: 5.3% to 4.6%) in 1990–1999, 3.4% (95% CI: 3.5% to 3.2%) in 2000–2009, and 1.4% (95% CI: 1.6% to 1.2%) in 2010–2019. In contrast, for females, the AAPC increased by 4.6% (95% CI: 4.9% to 4.3%) in 1990–1999, 2.7% (95% CI: 2.9% to 2.6%) in 2000–2009, and 0.5% (95% CI: 0.6% to 0.2%) in 2010–2019.

### Ecological correlations

**Fig 2:**
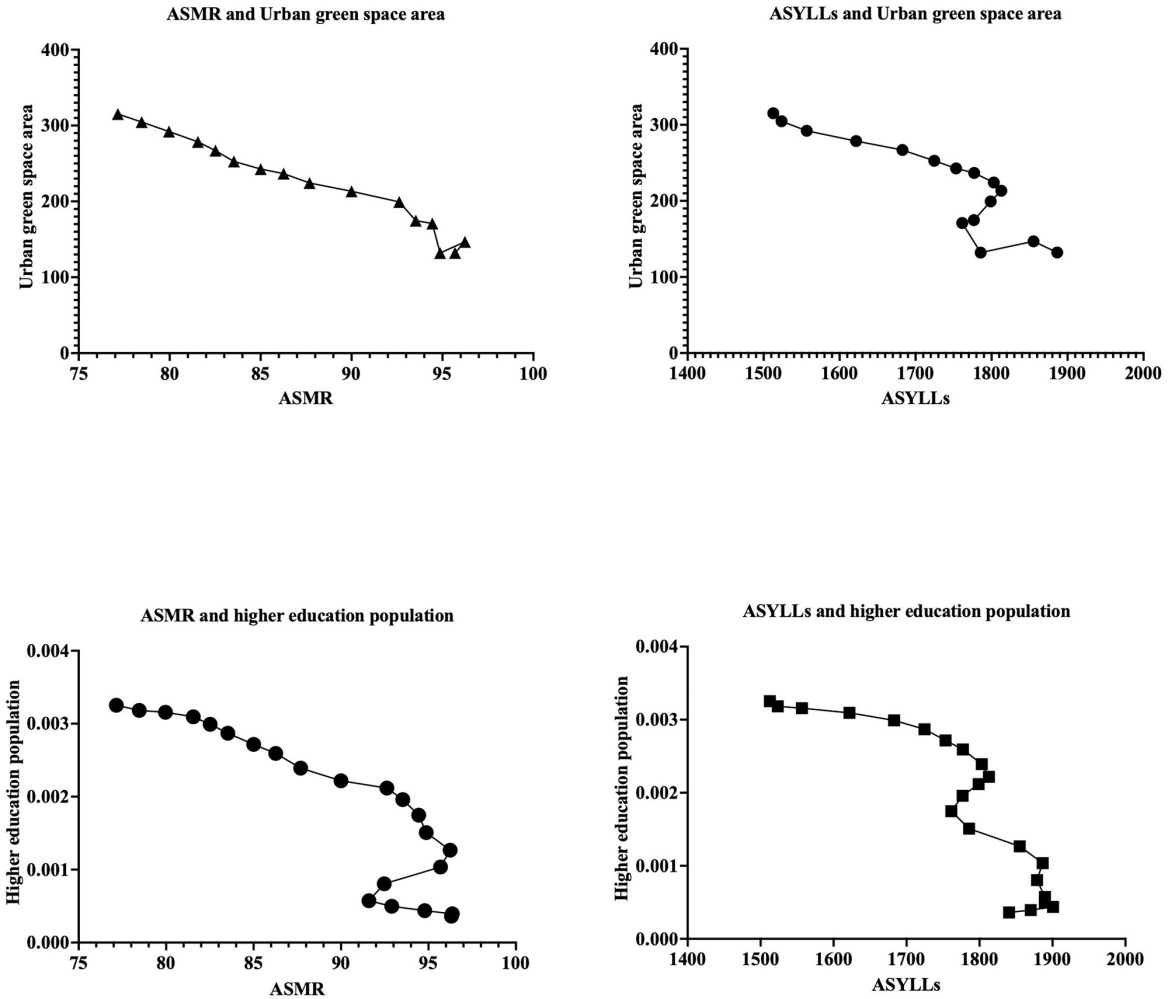
Association between mortality attributable to PM2.5 trends, urban green space area, and higher education proportions. ASMR = age-standardized mortality rates, ASYLLs = age-standardized years of life lost.

The Fig 2 shows a strong negative correlation between urban green space area and all causes ASMR attributable to PM2.5, with a Pearson’s correlation coefficient of -0.98 (95% CI: -0.99 to -0.95, p < 0.0001). This indicates that as the urban green space area increases, all causes mortality rate attributable to PM2.5 decreases. Similarly, there is a negative correlation between the proportion of the population with higher education and all causes mortality rate attributable to PM2.5, with a Pearson’s correlation coefficient of -0.88 (95% CI: -0.95 to -0.72, p < 0.0001). For YLLs, the results were similar.

## Discussion

### Main findings

We investigated the trends in disease burden attributable to ambient particulate matter pollution in China from 1990 to 2019. Our main findings include a slight increase in the ASMR for all causes attributed to PM2.5 from 1990 to 2019. During the same period, the ASMR for chronic obstructive pulmonary disease significantly decreased, while the ASMR for diabetes mellitus increased. We also observed increases in YLLs for some diseases, such as diabetes mellitus and tracheal, bronchus, and lung cancer. Furthermore, our analysis revealed strong negative correlations between urban green space area, the proportion of the population with higher education, and the all-cause ASMR attributable to PM2.5.

### Interpretation of main results

The observed increase in the ASMR for all causes attributed to PM2.5 may be due to various factors, including rapid urbanization, industrialization, and increased vehicular emissions in China over the past three decades [16]. These factors have contributed to a rise in air pollution levels, leading to a higher disease burden associated with PM2.5 exposure. The significant decrease in the ASMR for COPD, on the other hand, may be attributed to improved access to healthcare, early detection, and better management of the disease [17, 18]. Public health initiatives and advancements in medical treatments have likely played a role in reducing the mortality rates associated with COPD. Conversely, the increase in the ASMR for diabetes mellitus could be due to the growing prevalence of obesity, sedentary lifestyles, and unhealthy dietary habits in the Chinese population [19–21]. The rapid economic development and urbanization in China have led to changes in dietary patterns, with an increased consumption of high-calorie, processed foods and a decrease in physical activity. These lifestyle changes have contributed to a higher risk of developing diabetes mellitus, which in turn has increased the disease burden associated with PM2.5 exposure. Furthermore, the rise in ASMR for diabetes mellitus and other non-communicable diseases may also be influenced by an aging population, as the risk of developing these conditions increases with age [12, 22]. As China’s population continues to age, it is essential to implement effective public health strategies to address the growing burden of non-communicable diseases and mitigate the adverse effects of PM2.5 exposure on human health.

Based on our study findings, we observed a significant negative correlation between urban green space area and all-cause and cause-specific mortality rates attributable to PM2.5 in China. The negative correlation coefficient of -0.98 for urban green space area indicates a strong association, suggesting that an increase in urban green spaces is associated with a decrease in mortality rates related to PM2.5 pollution. Additionally, we found a negative correlation (-0.88) between the proportion of the population with higher education and all-cause mortality rates attributable to PM2.5. This implies that a higher proportion of individuals with higher education is associated with lower mortality rates related to PM2.5 pollution. Education may play a role in increasing awareness of health risks, promoting healthier behaviors, and facilitating access to resources that contribute to overall well-being.

One important confounding factor to consider is socioeconomic status, which is known to be associated with both the exposure to PM2.5 and health outcomes. Socioeconomic disparities, such as differences in housing conditions, income levels, and access to healthcare resources, could contribute to variations in mortality rates and potentially confound the associations observed in our study. Additionally, individual behaviors and lifestyle factors can serve as confounders in our analysis. Variables such as smoking habits, dietary patterns, physical activity levels, and healthcare-seeking behaviors may influence both PM2.5 exposure and mortality rates. These factors should be taken into account when interpreting the associations between urban green space area, education, and PM2.5-related mortality. While we did not directly control for these confounding factors in our study, it is important to acknowledge their potential impact on the observed associations.

### Comparison with other studies

Our findings are consistent with previous studies that have reported increasing trends in disease burden attributable to ambient particulate matter pollution in China [12, 22]. In particular, the observed increase in ASMR for diabetes mellitus is in line with the findings of a recent study that reported a positive association between PM2.5 exposure and the risk of diabetes [23]. Additionally, our results on the negative correlations between urban green space area, the proportion of the population with higher education, and the all-cause ASMR attributable to PM2.5 are supported by previous research highlighting the potential beneficial effects of green spaces on air quality and health [24, 25].

### Implications of the study

Our study has several implications for public health policy and practice in China. First, the increasing trends in disease burden attributable to ambient particulate matter pollution underscore the need for effective air pollution control measures, such as stricter emission standards, promotion of clean energy, and improved public transportation systems. Second, the observed negative correlations between urban green space area, the proportion of the population with higher education, and the all-cause ASMR attributable to PM2.5 suggest that urban planning and educational interventions could play a crucial role in mitigating the health impacts of air pollution in China.

### Strengths and limitations

The strengths of our study include the use of a comprehensive dataset covering a 30-year period and the application of advanced statistical methods to assess the trends in disease burden attributable to ambient particulate matter pollution. However, our study also has some limitations. First, the ecological study design precludes causal inferences and may be subject to ecological fallacy. Second, the reliance on secondary data sources may have led to potential biases in the estimation of ASMR and YLLs. Third, our analysis did not account for potential confounders, such as socioeconomic status and lifestyle factors, which could have influenced the observed associations. Forth, since we utilized GBD data, it only provides information at the national level in China and does not include data for different provinces or regions. This restricts our ability to compare and analyze disease burden trends between different areas.

## Conclusion

In conclusion, our study revealed increasing trends in disease burden attributable to ambient particulate matter pollution in China from 1990 to 2019, particularly for diabetes mellitus and tracheal, bronchus, and lung cancer. The observed negative correlations between urban green space area, the proportion of the population with higher education, and the all-cause ASMR attributable to PM2.5 highlight the importance of urban planning and educational interventions in mitigating the health impacts of air pollution. Further research is needed to explore the underlying mechanisms and identify effective strategies for reducing the disease burden associated with ambient particulate matter pollution in China.

## Supporting information

S1 Table Source data. (XLSX)

S2 Table APC and AAPC for all causes and cause specific mortality attributable to ambient particulate matter pollution in China. (XLS)

S1 Checklist STROBE statement—Checklist of items that should be included in reports of *cross-sectional studies*. (DOCX)
